# Enhancing end of life care on general internal medical wards: the 3 Wishes Project

**DOI:** 10.1186/s12904-023-01133-4

**Published:** 2023-02-14

**Authors:** Julie C. Reid, Brittany Dennis, Neala Hoad, France Clarke, Rajendar Hanmiah, Daniel Brandt Vegas, Anne Boyle, Feli Toledo, Jill C. Rudkowski, Mark Soth, Diane Heels-Ansdell, Andrew Cheung, Kathleen Willison, Thanh H. Neville, Jason Cheung, Anne Woods, Deborah Cook

**Affiliations:** 1 Department of Health Research Methods, Evidence, and Impact, Faculty of Health Sciences, McMaster University, McMaster University Medical Centre, 1280 Main Street West, 2C Area, Hamilton, ON L8S 4K1 Canada; 2 Department of Medicine, Faculty of Health Sciences, McMaster University, McMaster University Medical Centre, 1280 Main Street West, 2C Area, Hamilton, ON L8S 4K1 Canada; 3 Department of Critical Care, St. Joseph’s Healthcare Hamilton, 50 Charlton Avenue East, Hamilton, ON L8N 4A6 Canada; 4 Department of Medicine, St. Joseph’s Healthcare Hamilton, 50 Charlton Avenue East, Hamilton, ON L8N 4A6 Canada; 5 Division of Palliative Care, Department of Family Medicine, Faculty of Health Sciences, David Braley Health Sciences Centre, McMaster University, 100 Main Street West, 6th Floor, Hamilton, ON L8P 1H6 Canada; 6 Department of Spiritual Care, St. Joseph’s Healthcare Hamilton, 50 Charlton Avenue East, Hamilton, ON L8N 4A6 Canada; 7 School of Nursing, Faculty of Health Sciences, Health Sciences Centre, McMaster University, 1280 Main Street West, Hamilton, ON L8S 4K1 Canada; 8 Department of Medicine, Division of Pulmonary & Critical Care, University of California Los Angeles, Los Angeles, CA 90095 USA

**Keywords:** End-of-life, Palliative care, Acute care, Compassionate care, Patient-centered care, Empathy

## Abstract

**Background:**

Initially developed in the intensive care unit (ICU) at St. Joseph’s Healthcare Hamilton (SJHH) the 3 Wishes Project (3WP) provides personalized, compassionate care to dying patients and their families. The objective of this study was to develop and evaluate 3WP expansion strategies for patients cared for on General Internal Medicine (GIM) wards in our hospital.

**Methods:**

From January 2020-November 2021, we developed a phased, multicomponent approach for program expansion. We enrolled patients on the GIM wards who had a high probability of dying in hospital, then elicited, implemented, and documented wishes for them or their families. Data were analyzed descriptively.

**Results:**

From March 2020 to November 2020, we implemented staff education and engagement activities, created an Expansion Coordinator position, held strategic consultations, and offered enabling resources. From March 2020 to November 2021, we enrolled 62 patients and elicited 281 wishes (median [1^st^, 3^rd^ quartiles] 4 [4, 5] wishes/patient). The most common wish categories were personalizing the environment (67 wishes, 24%), rituals and spiritual support (42 wishes, 15%), and facilitating connections (39 wishes, 14%). The median [1^st^, 3^rd^] cost/patient was $0 [0, $10.00] (range $0 to $86); 91% of wishes incurred no cost to the program.

**Conclusions:**

The formal expansion of the 3WP on GIM wards has been successful despite COVID-19 pandemic disruptions. While there is still work ahead, these data suggest that implementing the 3WP on the GIM wards is feasible and affordable. Increased engagement of the clinical team during the pandemic suggests that it is positively received.

**Supplementary Information:**

The online version contains supplementary material available at 10.1186/s12904-023-01133-4.

## Introduction

Optimal end-of-life care for hospitalized patients is challenging. The majority of Canadians die within a healthcare institution cared for by clinicians [1, 2]. However, most people would prefer to die in their home, comforted by family and friends [3]. Many initiatives designed to improve end-of-life care have been introduced in clinical settings [4], but often fall short with respect to patient autonomy, effective communication, and care that is concordant with patient values [5, 6].

One intervention to honor what matters most for dying patients and enhancing relationships among patients, families, and clinicians is the 3 Wishes Project (3WP) [7]. Originally developed to improve the experience of dying in the intensive care unit (ICU), the 3WP involves eliciting and facilitating final wishes for dying patients and their loved ones. This intervention promotes legacy-building, while enabling clinicians to support and comfort families at the end-of-life [7–9]. A binational, multi-center evaluation of the 3WP demonstrated value from the perspectives of families, clinicians, and hospital managers. This study also documented sustainability as evidenced by participating sites continuing the program after study completion, and the need for minimal resources (mean cost/wish of $5.19) [8, 10]. Adaptations of the 3WP have been described in North American academic centers [8–10], Canadian community hospitals [11, 12], an oncology ward in the United States [13], and for individuals experiencing homelessness [14]. The 3WP transitioned from a research study to ongoing day-to-day practice in the ICU at the founding site, and continues to inspire acts of compassion [8, 15, 16], preserve patient dignity, ease family grief, and provide closure for clinicians [9].

To explore expansion of the 3WP to the General Internal Medicine (GIM) hospital wards in our institution, we pilot tested the 3WP by implementing 117 wishes for 23 dying patients between January 2017 and March 2020 [17]. Facilitating connections, providing care to families, and personalizing the environment were the most common wish categories. Affordability was supported by a mean cost/patient of $16, while 85% of wishes incurred no cost to the program [17]. In the latter parts of the pilot phase, clinician interest and hospital leadership commitment drove the development of a more structured expansion. As the pilot phase occurred over a protracted period and was enabled by the 3WP ICU team, we developed this expansion phase to facilitate more autonomy for staff on the GIM wards to initiate and implement the 3WP. Our objective therefore was to develop and describe our 3WP implementation activities and evaluate the strategies in terms of staff engagement, patient enrolment and wish details for patients on the GIM wards in our hospital.

## Methods

### Design and setting

This phased, multicomponent cohort study was conducted at St. Joseph’s Healthcare Hamilton (SJHH), a large academic teaching hospital from January 2020 to November 2021. We engaged the 4 GIM wards, including 123 beds across 3 clinical teaching units with the capacity for telemetry monitoring and 1 medical step-down unit that is a designated level 2 critical care unit capable of providing life support excluding invasive mechanical ventilation. While the hospital does not have a specific palliative care ward, it has an interprofessional palliative care consultation service, and some teaching units have a dedicated palliative suite.

### Participants

#### Patients

We invited patients and families to participate in the 3WP who were ≥ 18 years of age and had a high likelihood of dying during the hospital stay as judged by the attending physician.

#### Staff

All clinical staff, including nurses, physicians, etc. on these wards were invited to contribute to the 3WP expansion, which involved identifying dying patients, and participating in wish elicitation and implementation.

### 3WP Intervention

Staff, patients, and families or friends could be involved in any stage of the wish process; engagement emerged organically. Aligned with our pilot work, we recorded patient data and wish characteristics, including who made the wish for the patient (i.e., patient, family or friend, clinician), who implemented it, and the cost. Additional file 1: Appendix 1 (section A1.1) details steps in the 3WP initiation and progression for dying patients.

### 3WP expansion methods

#### Expansion Coordinator

A part-time 3WP Expansion Coordinator role was developed to facilitate the expansion. An ICU registered nurse (RN) with substantial 3WP experience was recruited to this role (NH).

#### Interprofessional staff engagement

We used 4 implementation strategies to engage interprofessional staff: 1) off-site orientation sessions, 2) in-hospital drop-in sessions, 3) in-person small group ward-based huddles, and 4), video-conferenced in-services including a recorded 3WP description followed by a synchronous interactive question and answer period.

#### Learner engagement

We engaged learners to foster the 3WP philosophy among their cohort, including 1) Spiritual Care residents (we presented the 3WP during their orientation) 2) Internal Medicine residents (we dedicated an academic half day to the 3WP and oriented new residents at the start of their GIM rotation). A Resident Champion (BD) provided experiential teaching and role modelling on eliciting and facilitating wishes (and was available to answer questions from residents throughout the expansion period).

#### 3WP resource group

We established a 5-member 3WP Resource Group including the 1) Expansion Coordinator (NH), 2) Resident Champion (BD), 3) 3WP Research Coordinator (FJC, who also facilitated wish elicitation and implementation), 4) GIM Spiritual Care clinician (FT) with extensive 3WP experience, and 5) a new 3WP Post-Doctoral Fellow (JCR) employing health services approaches to enhance and evaluate the expansion.

#### Strategic consultations for training and support

To facilitate implementation, we consulted bedside nurse champions and nurse educators on each ward, GIM physician champions with expertise in end-of-life care, and the palliative care team, to co-design strategies for their respective roles and their colleagues.

#### Funding

The study team secured two internal grants totalling $15,000 to provide consumables for wishes, refreshments for staff engagement events, data collection and analyses, and anticipated submission of posters and manuscripts for publication. To further support the program, assisted by ICU and GIM colleagues, ICU survivors, and 3WP families, the study team also engaged in fundraising for a hospital foundation event.

#### Wish supplies

We provided each GIM ward with items including resource binders, colourful handmade blankets, flameless candles, Bluetooth speakers, and supplies for making keepsakes (e.g., for fingerprint keychains, word clouds [18], etc.).

#### Informational resources

We created written material in paper and digital formats, (i.e*., 3WP Frequently Asked Questions)* and information for the GIM and Nursing Education Newsletters, along with posters highlighting successes-to-date (adapted from 3WP At-A-Glance posters developed at University of California, Los Angeles (UCLA)) [19], and sent periodic email correspondence to allied health and clinical staff.

#### Chart documentation

For documenting 3WP in patients’ charts, we developed a note template for the electronic medical record (EMR) in Epic™ (Additional file 1: Appendix 1, section A1.2, E-Fig. 1). This progress note included the purpose of the 3WP, persons involved in the conversations, and wish details (e.g., what is the wish, wished by whom, and implemented by whom).

### Ethics

This study was reviewed by the Hamilton Integrated Research Ethics Board and deemed it a quality improvement initiative. As such, they waived the need for written informed consent for implementing acts of compassion through the 3 Wishes Project. Anonymized data collection was in accordance with this quality improvement initiative.

### Statistical analyses

We computed descriptive statistics of continuous variables as means and standard deviations, or medians and 1^st^ and 3^rd^ quartiles if data were skewed. We calculated counts and percentages for categorical variables. We descriptively compared wish characteristics between this expansion phase and the pilot study. Additionally, we descriptively compared characteristics of patients with and without COVID-19 and their wishes, tracking visiting restrictions, implementation activities, and enrolment rates before and during the pandemic.

## Results

### Timelines for 3WP Expansion

**Fig. 1:**
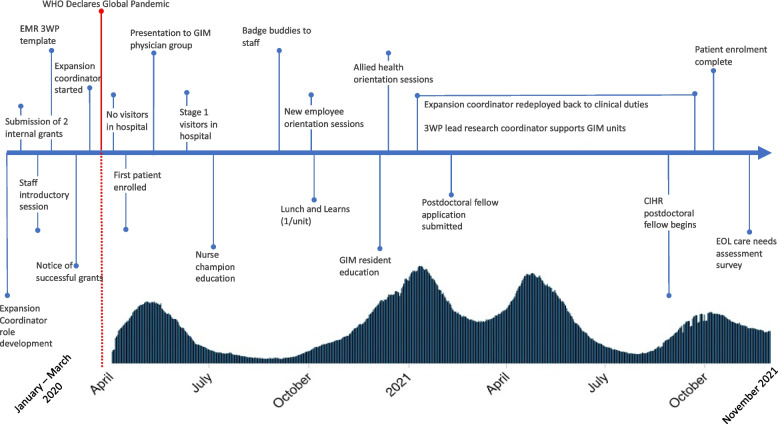
Timeline of 3WP expansion activities from January 2020 – November 2021. Legend: This figure shows the timeline of formal 3WP expansion activities in the context of the various waves of the COVID-19 pandemic in dark green pattern from March 2020 to November 2021 in Ontario, Canada (showing patterns of hospitalized patients) [20, 21]. Abbreviations: EMR – electronic medical record; WHO – World Health Organization; GIM – general internal medicine; CIHR – Canadian Institutes of Health Research; EOL – end-of-life care

Figure 1 shows a timeline of expansion events. In early January 2020, preparatory activities were underway with the development of the Expansion Coordinator role, submission of internal grants to support expansion activities, and an off-site introductory event. Please see Additional file 1: Appendix 1 (section A1.3) for further detail about this and 3WP Resource Group Activities.

### 3WP resource group activities

Between May 2020 and November 2020, the 3WP Expansion Coordinator facilitated orientation activities including presentations, webinars, orientations, and drop-in sessions (Additional file 1: Appendix 1, section A1.3, E-Table 1). She also conducted weekly check-ins with each ward for the first 6 months, which were paused when the pandemic burden increased. The Expansion Coordinator and the Research Coordinator also created a ‘badge buddy’ adapted from 3WP ‘badge buddies’ developed at the UCLA to give to interested nursing staff (Additional file 1: Appendix 1, section A1.4, E-Fig. 2; T. Neville, personal communication, Aug. 10, 2019). As of September 2021, co-funded by Canadian Institutes of Health Research, the hospital, and the Academic Critical Care Research Office, the 3WP Post-Doctoral Fellow assisted with the 3WP expansion and developed additional health services approaches to enhance end-of-life care practices on the GIM wards.

### 3WP patients and wish characteristics

The first patient enrolled with assistance from the Expansion Coordinator was on March 3, 2020, and the last patient for whom data were collected in this phase was on November 8, 2021. We enrolled 62 patients in the 3WP from the 4 GIM wards. Most were female (53.2%), and the mean age was 78.6 years. Three patients (4.8%) died after being transferred home (*n* = 2) or to a continuing care facility (*n* = 1). Table 1 summarizes patient demographics and characteristics of their hospital stay. Patients are continuing to receive care aligned with the 3WP as initiated by staff on the wards and supported by the 3WP Resource Group as needed.

**Table 1:** Patient demographics and hospital stay characteristics

Characteristic	Value
**Patient demographics**	
Age in years, mean (SD)	78.6 (11.7)
Female sex, n (%)	33 (53.2)
Race/ethnicity, n (%)	
White	55 (88.7)
Asian	4 (6.5)
Indigenous	3 (4.8)
Spiritual belief, n (%)	
Christian^a^	37 (59.8)
Spiritual	4 (6.5)
Buddhist	1 (1.6)
Jewish	1 (1.6)
Sikh	1 (1.6)
No religion	12 (19.4)
Unknown	6 (9.7)
**Hospital stay characteristics**	
Code status documented on hospital admission, n (%)	
Full code (plans for advanced life support if necessary)	26 (41.9)
No code (plan not to intubate or perform cardiopulmonary resuscitation)	36 (58.1)
Location at enrolment, n (%)	
GIM ward	55 (88.7)
Medical step-down ward	6 (9.7)
Emergency room	1 (1.6)
Relevant consultations, n (%)	
Spiritual care	42 (67.7)
Social work	41 (66.1)
Palliative care	40 (64.5)
Length of stay, median days [1^st^, 3^rd^ quartiles]	
Hospital admission to enrolment in 3WP^b^	10 [4, 16]
Enrolment in 3WP to death^c^	3 [1, 8]
Hospital admission to death^c^	16 [7, 26]
Died in hospital, n (%)	59 (95.2)
Family or friend(s) present at time of death in hospital, yes, n (%)	41 (69.5)
Inside patient’s room	39 (95.1)
Outside patient’s room	1 (2.4)
Via virtual technology	1 (2.4)

*Abbreviations*: *SD* Standard deviation, *ER* Emergency room, *EOL* End of life, *3WP* 3 Wishes Project

*Legend*

^a^ Christian denominations represented are: Catholic (*n* = 17, 27.4%), United (*n* = 5, 8.1%), Anglican (*n* = 3, 4.8%), Lutheran (*n* = 3, 4.8%), Jehovah’s Witness (*n* = 2, 3.2%), Protestant (*n* = 2, 3.2%), Baptist (*n* = 1, 1.6%), Eastern Orthodox (*n* = 1, 1.6%)

^b^ includes all 62 patients;

^c^ only includes the 59 patients who died in hospital

Enrolment in the 3WP was most often initiated by a bedside nurse (16, 25.8%), followed by a palliative care clinician (13, 21.0%), an attending physician (10, 16.1%), social worker (5, 8.1%), and spiritual care clinician (5, 8.1%). Patients were admitted to the hospital for a median of 10 days prior to enrolment; the median time from enrolment to death was 3 days.

**Fig. 2:**
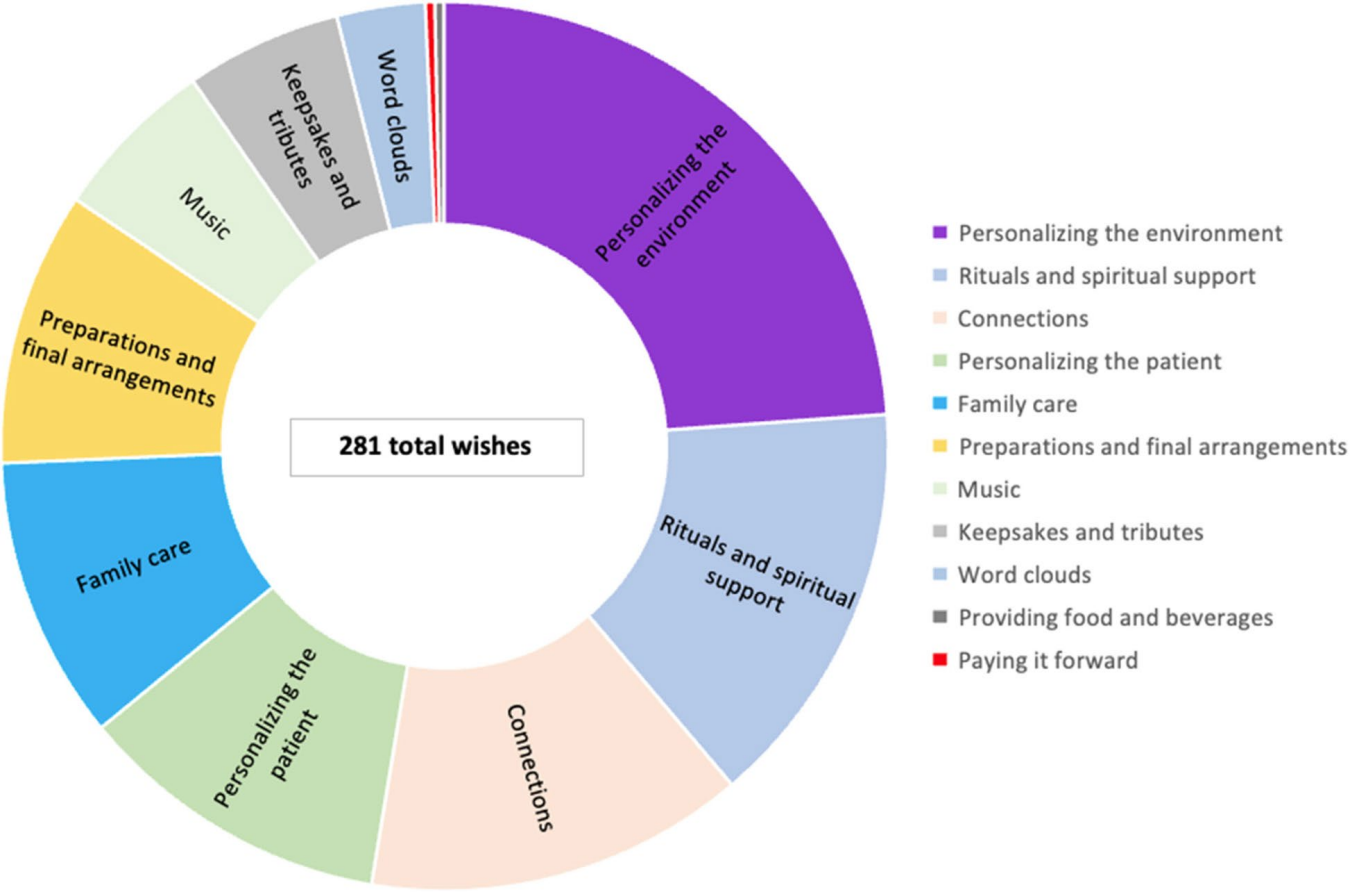
Proportion of wishes for 62 dying patients distributed amongst 11 wish categories. Legend: Wish categories: personalizing the environment (*n* = 67, 23.8%), rituals and spiritual support (*n* = 42, 14.9%), facilitating connections (*n* = 39, 13.9%), personalizing the patient (*n* = 32, 11.4%), family care (*n* = 29, 10.3%), preparations and final arrangements (*n* = 28, 10.0%), music (*n* = 17, 6.0%), keepsakes and tributes (*n* = 16, 5.7%), word clouds (*n* = 9, 3.2%), providing food and beverages (*n* = 1, 0.4%), paying it forward (*n* = 1, 0.4%)

There were 281 wishes documented for the 62 patients with a median [1^st^, 3^rd^ quartiles] number of wishes/patient of 4 [4, 5]. Figure 2 shows the number of wishes for each of the 11 wish categories (category descriptions have been published previously [17]). Wishes were most commonly made by family or friends (145 wishes, 51.6%), followed by clinicians (89 wishes, 31.7%). Nearly half of patients (*n* = 30, 48.4%) engaged in some aspect of the wish process (i.e., making or implementing); direct patient participation informed the selection of 73 wishes (26.0%). Patients most requested to taste a favorite food or drink, go outside, or have their family pet visit (personalizing the patient category, 24.7%), while family and friends most often wished to facilitate connections (28.3%), and clinical teams wished to personalize the environment (41.3%) including providing a handmade blanket or transferring the patient to a private room or palliative care suite when possible.

Nearly all wishes (276, 98.2%) were implemented; only 5 wishes were not possible for medical (*n* = 3) or logistical (*n* = 2) reasons. Most wishes (*n* = 268, 95.4%) were made ante-mortem but 13 wishes (4.6%) were made post-mortem. Post-mortem wishes included keepsakes (*n* = 9, e.g., word clouds and fingerprint keychains), prayers (*n* = 2), playing a favorite radio station at the bedside (*n* = 1), and donating to the 3WP (*n* = 1). Wishes were most often implemented by nurses (90 wishes, 32.6%), followed by family (46 wishes, 16.7%), and spiritual care clinicians (46 wishes, 16.7%). Others implementing wishes were either hospital-based (e.g., charge nurse, security staff, medical learners (resident and student), recreational therapist, Infection Prevention and Control staff, and nursing and hospital administrators) or community-based (e.g., primary care physician, Indigenous elder, priest).

The median [1^st^,3^rd^] cost per wish was $0 [0,0] and the median [1^st^, 3^rd^] cost per patient was $0 [0, $10.00]. Seventy-two wishes (26.1%) were donated (*n* = 70) or discounted (*n* = 2), and 251 wishes (90.9%) incurred no additional cost to the program (i.e., wish cost = $0).

### Influence of the pandemic

During the pandemic, SJHH cared for patients with COVID-19 in several areas of the hospital, including the ICU, a dedicated COVID ward, and on the GIM wards as additional beds were needed to care for patients under surge conditions. Because of the influx of patients with COVID-19 and ongoing high patient volumes, the Expansion Coordinator was redeployed back to clinical nursing duties in the ICU between December 2020 and October 2021 (Fig. 1). Thereafter, the 3WP Research Coordinator supported the wards by coaching staff, stocking supplies, and facilitating wishes such as keepsakes as requested.

Strict visiting restrictions were implemented in March 2020. Under special circumstances such as end-of-life, brief, single visits by one or a limited number of family or friends were sometimes permitted. By June 2020, visitation became less restrictive, particularly for dying patients, but this was not universal for patients with COVID-19 infection. Restrictions fluctuated in terms of how many visitors could attend, if they could enter the patient’s room, and the duration of the visit depending on location, shifts, clinician discretion, and pandemic burden. For 7 patients (11.3% of total cohort) with COVID-19 enrolled during this study, 29 wishes were implemented. Only 3 of these patients (42.9%) had family or friends present at the time of death compared to 73.1% of patients without COVID. Compared to patients without COVID, those with COVID were more likely to have wishes facilitated by clinicians only (41.4% vs. 18.7%, respectively).

### Comparison to pilot wish characteristics

There were differences in terms of wish elicitation and wish categories between patients in the pilot phase and in this expansion study (Table 2). For example, more clinical team members were engaged in wishes (28.5% in this expansion compared to 11.1%). Given limited family and friend presence at the bedside during the expansion phase, clinician wishes for family care were less common than in the pilot (18.8% compared to 46.2%). Patient wishes for food and beverages occurred less commonly than in the pilot (0% compared to 6.3%). Clinician wishes for rituals and spiritual support were higher in this expansion period than in the pilot (16.4% compared to 3.1%).

**Table 2:** Percent of patient, family, and clinical team wishes in the pilot and expansion phase

Wish Category	Patient-wished		Family/friend-wished		Clinical team-wished	
	**Pilot**	**Expansion**	**Pilot**	**Expansion**	**Pilot**	**Expansion**
Facilitating connections	31.3	17.8	32.3	28.3	0	12.5
Providing food and beverages	6.3	0	1.5	0	7.7	0
Personalizing the environment	12.5	13.7	16.9	13.8	15.4	41.3
Personalizing the patient	25.0	24.7	1.5	6.9	15.4	7.5
Music	3.1	6.8	9.2	6.2	0	7.5
Family care	3.1	0	4.6	9.0	46.2	18.8
Rituals and spiritual support	3.1	16.4	13.8	20.7	0	8.8
Preparations and final arrangements	15.6	20.5	3.1	10.3	0	2.5
Word clouds	0	0	16.9	4.8	7.7	1.3
Keepsakes and tributes	3.1	1.4	6.2	6.9	0	7.5
Paying it forward	0	0	3.1	0.7	0	0
**Proportion of wishes overall**						
	27.4	26.9	55.6	51.7	11.1	28.5

## Discussion

This phased, multicomponent expansion of the 3WP on GIM wards has been feasible even with the disruption of the COVID-19 pandemic. We elicited 281 wishes from 62 patients over 20-months and fulfilled 98% of them. Wishes offer healthcare workers the opportunity to recognize a patient as an individual and engage in acts of kindness during the dying process. Our findings confirm our pilot work suggesting the feasibility and affordability of the 3WP for medical patients. Engagement of diverse members of the interprofessional clinical team and others such as security staff, Infection Prevention and Control staff, and community physicians demonstrates its positive reception.

Nearly a quarter of wishes in this expansion phase were for personalizing the environment, aligned with multicenter findings in the ICU setting [8] where 15% of all wishes were in this category (e.g., flowers, decorations, personal photos for the room). In contrast, facilitating connections was the commonest category in our pilot phase [17]. Hospital visitation was severely restricted during the pandemic, which may explain the increased focus on environmental enhancement for patients when their family or friends could not be present. Community engagement increased in the expansion phase, perhaps heightened due to the pandemic, reflected in 25% of wishes being donated compared to 16% donated in the pilot [17] and 19% in the multicenter ICU study [8]. Across the ICU studies [7, 8, 10], pilot phase [17], and this expansion study, the median cost of $0/wish underscores the program’s ongoing affordability. Notably, this cost data reflects consumables related to wishes and does not include costs for staff exposure and engagement, which occurred during shifts in the hospital, and some additional invitational events for further training for those interested, sometimes during a shift and sometimes on their time off. The dedication of our GIM teams, support from the 3WP Resource team, and two modest local grants as described in the Methods section enabled the program’s start-up on the wards.

Other notable differences between this study and our pilot study included initiation of the 3WP by bedside nurses more than 25% of the time compared to 13% in the pilot phase [17], in keeping with increased staff engagement, and is encouraging for program sustainability. Correspondingly, physicians enrolled 16% of patients in this expansion period compared to nearly 40% in the pilot study, perhaps related to deliberate physician leadership in the exploratory pilot phase and an intentional shift in the expansion phase to foster experience among bedside nurses and more diverse clinical team members who fulfilled 29% of wishes compared to 11% in the pilot study.

The onset of the COVID-19 pandemic coincided with this 3WP expansion, impacting several aspects of this project. First, strict policies on gathering and physical distancing limited face-to-face staff interactions. This necessitated a shift to virtual training strategies, and lost opportunities for the 3 Wishes Resource Group to connect with staff on site offering encouragement and support. Policies prohibiting families or friends from being at the bedside was manifest in those individuals implementing fewer wishes. Additionally, members of the 3WP Resource Group, including the Expansion Coordinator and Resident Champion who were practicing clinicians were redeployed, curtailing some development activities and enrolment opportunities.

The pandemic has also borne new ways (or revitalized old ways) of doing things. Aspects of the 3WP implemented throughout the pandemic included clinician-led acts of kindness such as relaying family messages during final moments [22]. Technology-assisted virtual visits emerged as an alternative way to help maintain contact with families. And while both clinicians and families described challenges such as inequitable access to technology, variable technological literacy, and the time required to facilitate these connections [22, 23], videoconferencing has provided some comforting connection and reassurance to families when their presence was prohibited [24]. While not a formal aspect of the expansion, citizens in our community were motivated to support patients during the pandemic in the form of knitted, crocheted, or quilted handmade blankets and paired keepsake hearts. Volunteers who were disallowed in the hospital dropped off crafted keepsakes at the front entrance. The 3WP Research Coordinator also delivered supplies and picked up finished crafts in the community as needed throughout the pandemic.

Two national consensus statements outline complementary overarching principles to achieve ‘a good death’. These include open, honest, and patient-centered communication, addressing individual preferences and needs during the dying process, adequate symptom management, and supporting patient’s families before and after a patient’s death [25, 26]. A recent meta-synthesis identified six common themes from patients and families: expert care, effective communication and shared decision-making, respectful and compassionate care, adequate environment for care, family involvement, and financial affairs [27]. However, these ideals are often deficient in institutional settings, particularly on hospital wards. For example, Rolnick et al. (2020) evaluated surveys from family members or loved ones of 28,062 decedents in the ICU, on a ward, or having been care for in both settings. The care was rated significantly better overall for those in the ICU compared to a ward, including preference-concordant care, symptom management, staff treatment with kindness and respect, emotional support before and after death, and spiritual support [28]. These differences in care could reflect higher mortality among critically ill patients, and higher nurse:patient ratios in the ICU, affording more opportunity to foster relationships and enhance staff experience caring for dying patients.

Our study has limitations. Initially the study team including the 3WP Resource Group identified patients for enrolment, such that decedents were non-consecutive as we gradually expanded the 3WP. The modest sample size reflects the single center design and impact of the pandemic. We were unable to accurately identify the total number of patients cared for and the total number who died under the GIM service for the duration of the study due to medical patients being cared for off-service on non-GIM wards (surgical wards or the ICU) for part or all of their hospital stay to accommodate all patients with and without COVID in the hospital. The population of predominantly white patients of Christian faith is relatively homogeneous, and staff diversity was not recorded. Wish preferences may differ in more multicultural, diverse communities. We did not collect qualitative data to understand the perspectives of patients, families, or clinicians.

Our study also has strengths, including a staged interprofessional program expansion on 4 acute GIM wards. We describe the types of patients and wishes implemented, and a broad range of hospital staff who were involved in the program implementation and the care of dying patients. We continued the expansion despite the severe impact of the global pandemic on patients, staff, and resources, conducting studies like this one and coordinating several COVID-specific and non-COVID-specific studies for severely ill medical patients needing critical care [29]. Our multimethod approach could serve as a template for other health services interventions in this setting.

Since the study period ended, fulfilling wishes on the GIM wards has continued through the intentional work of nurses, physicians, and other staff along with the support of the 3WP Resource Group. This approach to practice is encouraging for continued uptake, and our results suggest feasibility, affordability, and positive signals for sustainability. The 3WP Resource Group is now developing a 3WP Volunteer Role within existing hospital volunteers. The Post-doctoral Fellow is conducting a larger project focused on broadly enhancing end-of-life care practices on the GIM wards, including a staff needs assessment and interventions to address the identified barriers, leverage facilitators, and address knowledge gaps.

End-of-life care is a crucial aspect of care which often falls short for those dying in hospital. The 3WP, aimed at honoring what matters most for dying patients and enhancing relationships among patients, families, and clinicians, seeks to improve the dying experience for both patients and their loved ones. While there is still work ahead to improve end-of-life practices, this study provides an important foundation for further work. The increased engagement of nurses and other clinicians provides an excellent foundation to enhance end-of-life practices that can further improve the dying experience for patients, families, and clinicians alike.

## Supplementary Information

**Additional file 1:** **Appendix 1.** Section A1.1 – 3 Wishes Project description. Section A1.2 – Example of 3WP clinical note. Section A1.3 – 3WP expansion results. Section A1.4 – 3WP badge buddy.

## Data Availability

Data will be available upon reasonable request from the corresponding author and approval by the study team.

## Abbreviations

3WP: 3 Wishes Project
CIHR: Canadian Institutes of Health Research
EMR: Electronic medical record
EOL: End-of-life
ER: Emergency room
GIM: General internal medicine
ICU: Intensive care unit
RN: Registered nurse
SD: Standard deviation
SJHH: St. Joseph’s Healthcare Hamilton
UCLA: University of California, Los Angeles
WHO: World Health Organization
