# Supplementary material for: Enhancing end of life care on general internal medical wards: the 3 Wishes Project
Source: BMC Palliat Care. 2023 Feb 14;22:11. doi: 10.1186/s12904-023-01133-4 (PMC9925934; doi:10.1186/s12904-023-01133-4)
Supplement: Supplementary file 1 — Additional file 1: Appendix 1. Section A1.1 – 3 Wishes Project description. Section A1.2 – Example of 3WP clinical note. Section A1.3 – 3WP expansion results. Section A1.4 – 3WP badge buddy. [file 12904_2023_1133_MOESM1_ESM.docx]

**Title:** Enhancing End of Life Care on General Internal Medical Wards: The 3 Wishes Project

**Authors:**

Julie C Reid, MSc PT, PhD

Brittany Dennis, MD, PhD

Neala Hoad, RN

France Clarke, RRT

Rajendar Hanmiah, MD

Daniel Brandt Vegas, MD, MHPE

Anne Boyle, MD

Feli Toledo, MDiv

Jill C Rudkowski, MD

Mark Soth, MD

Diane Heels-Ansdell, MSc

Andrew Cheung, MD

Kathleen Willison, RN, MSc

Thanh H Neville, MD, MSHS

Jason Cheung, MD

Anne Woods, MD

Deborah Cook MD, MSc

**Appendix 1**

| Section | Description | Page number |
| --- | --- | --- |
| A1.1 | 3 Wishes Project description | 2 |
| A1.2 | Example of 3WP clinical note | 3 |
| A1.3 | 3WP expansion results | 4 – 5 |
| A1.4 | 3WP badge buddy | 6 |

**A1.1 – 3 Wishes Project:**

The philosophy of the 3 Wishes encompasses the objectives of celebrating the patient’s life and providing a dignified death, humanizing the dying process, creating positive memories for the patient’s loved ones, and inspiring a deeper sense of meaning for clinicians. With that, there are no set rules on when to initiate or what specifically to do for patients and their loved ones, rather to determine what is important to them at that time and help facilitate those acts of kindness and compassion.

**Who is appropriate for 3 Wishes?**

There are some criteria that may prompt healthcare providers to consider a patient for 3 Wishes, including a >95% chance of dying during this hospital admission, or if a decision has been made to transition goals of care to ‘comfort measures’ only.

**When/how to initiate:**

When any healthcare provider identifies a patient that may be suitable for 3 Wishes (as above), they may initiate the conversation with the patient or family. It may be helpful to include any clinician that has an existing rapport with the patient or their loved ones.

The following are some suggested approaches to facilitate the conversation:

- Introduce yourself and acknowledge the difficulty of the situation
- Learn more about the patient – “we would like to learn more about [patient] and try to help during this difficult time. Can you tell me a bit about [patient]? How could we honor him/her? We want to do what we can to honor [patient] as well as support you through this
- Some probing questions could include:
- What did s/he like to do before becoming ill?
- What is most important to [patient]?
- What are [patient]’s favorite things, places, memories?
- Who are the other family members or loved ones we might meet?
- What do you need right now?
- Is there anything we can do for your family as you are here today?

**How to:**

Wishes are simple, meaningful, individualized expressions of a request or a need that can be fulfilled for a dying patient and/or grieving family member. Wishes give a voice and agency to patients and their families, and provide an opportunity to find meaning at the end of life. Calling them ‘wishes’ may be just giving a name to something that is already done as part of your practice or ‘standard of care’, for example, getting something to eat or drink for the patient.

**Wishes – the who, how, and what:**

Anyone who knows something about the patient can make a wish. Ideas for wishes tend to evolve organically from conversations with patients and their families. Often, an initial wish is offered by the clinical team (e.g., offering snacks, placing a comforting blanket on the patient, or providing extended time and privacy for visits) to start the conversation and thinking about wish possibilities.

Some examples of wishes include facilitating liberal visiting as able (in terms of hours and number of visitors), if able, move the patient to a single room or palliative care suite for privacy, allow family to bring in birthday or celebratory cakes or meals, celebrate the patient’s favorite holidays with decorations and music (e.g., Christmas or another holiday), provide a cozy non-hospital blanket for comfort, encourage families to bring in photos or other personal effects, allow the patient to dress in their own clothes if able, facilitate going outside, offer parking passes for family members after the patient dies, engage the Spiritual Care team to facilitate rituals and ceremonies, create keepsakes such as fingerprint keychains or word clouds.

**A1.2 – Clinical note ***this note represents a fictional patient for demonstration purposes only.


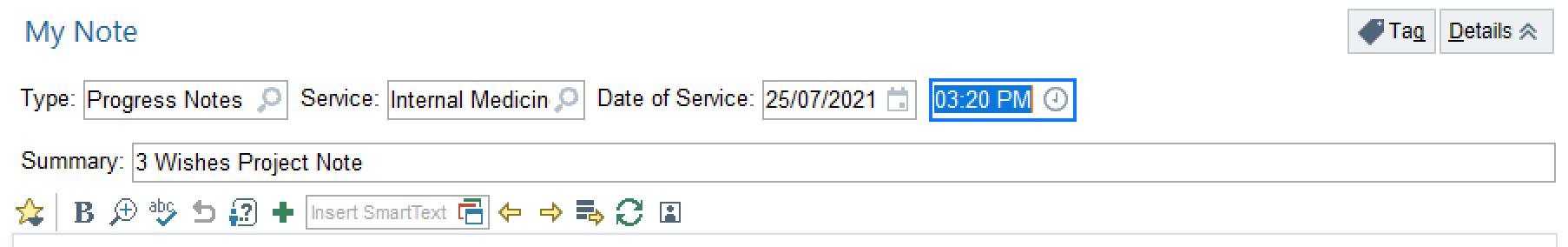

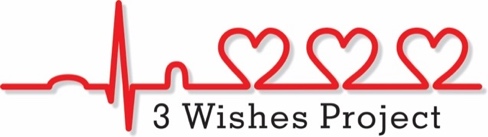


Date: July 25, 2021

Patient: 68-year-old male admitted to hospital on June 29^th^, 2021 for pneumonia.

The clinical team met with Mr. Black and his family and described the 3 Wishes Project (3WP). The goal is to have conversations with the patient (if able) and the family/friends (as available) to provide individualized end of life care, understand what would be meaningful to Mr. Black and his family at this time.

The project was initiated by: LT, bedside RN.

Information was obtained from: Janice, the daughter of Mr. Black.

The patient/family/friend/clinical staff have provided and facilitated the following wishes:

1. Humanizing the environment; Placed a colourful donated knitted blanket on his bed

Wished by: Bedside clinical staff

Implemented by: Bedside clinical staff

2. Rituals & Spiritual Support; Visit from Elder and gift of sage

Wished by: Indigenous community member

Implemented by: Indigenous Elder

3. Family care; Physician to complete employment form for Mr. Black’s wife’s

Wished by: Family

Implemented by: DS, Social Worker and JF, GIM physician

4. Preparations & Final Arrangements; Journal for Mr. Black to write messages to his family

Wished by: Patient

Implemented by: Family

5. Connections; Multiple family members and friends to have extended visits

Wished by: Patient and family/friends

Implemented by: MZ, Nursing Manager

6. Family care; Parking passes for family

Wished by: Bedside clinical staff

Implemented by: DS, Social Worker and 3 Wishes Team

Thank you for the opportunity to be involved in the care of Mr. Black and his family.

3 Wishes Project Team

**E-Figure 1.** EPIC clinical note for patient charts.

**A1.3 – 3WP Expansion Results**

**Timeline for the 3WP Expansion**

The off-site introductory event included presentations from the 3WP Resource Group, and question and answer periods to foster discussion and clarify approaches. This session was attended by the GIM Clinical Teaching Unit Director (RH), Medical Step-down Unit Head of Service (JR), bedside nurses, Nurse Managers, Volunteer Resources Director and Coordinator, Clinical Nurse Specialist (KW), a GIM-affiliated Spiritual Care Clinician (FT), and the Critical Care Director who represented hospital leadership as an expansion champion.

**3WP Resource Group Activities**

The expansion coordinator facilitated several orientation activities from May 2020 to November 2020.

**E-Table 1:** Expansion-coordinator facilitated orientation activities by target group.

| **Activity** | **Target group** | **Number of sessions** |
| --- | --- | --- |
| Presentation by 3WP physician collaborator (RH) | GIM staff physician group | 1 |
| Webinars | Select nurse champions from each ward | 2 |
| 3WP presentation | New employee orientation | 2 |
| Lunch and Learn drop-in sessions | All GIM staff | 4 |
| Orientation sessions | Allied team members (occupational therapy, recreational therapy, social work, spiritual care) | 6 |
| 3WP recorded presentation | Available to all staff by email link and posted on a main desktop computer on each ward | 1 |
| Orientation sessions | Nurse educators from each ward | 2 |

**A1.4 – 3WP Badge Buddy**


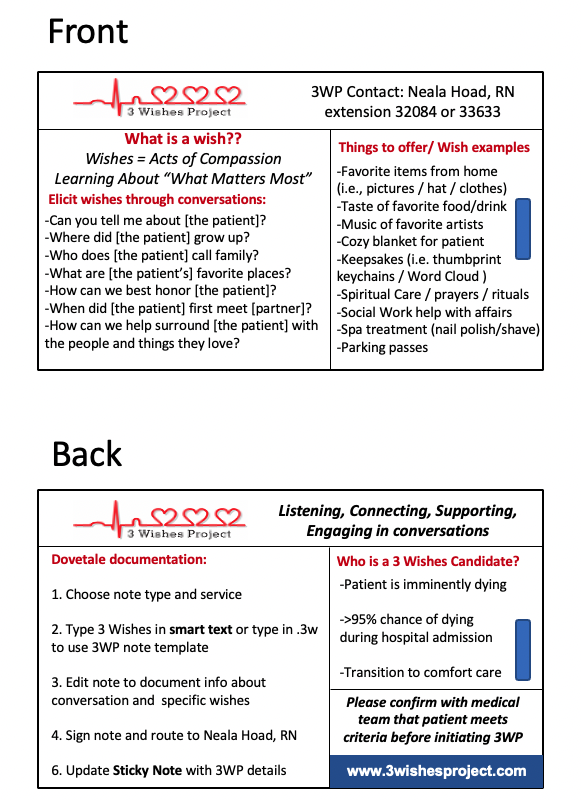


**E-figure 1.** Adapted 3WP badge buddy for nursing staff.
